# Strategies for resolving challenging psychedelic experiences: insights from a mixed-methods study

**DOI:** 10.1038/s41598-024-79931-w

**Published:** 2024-11-21

**Authors:** Maximillian J. Wood, Rosalind G. McAlpine, Sunjeev K. Kamboj

**Affiliations:** 1 Research Department of Clinical, Educational and Health Psychology, University College London, London, UK; 2 Clinical Psychopharmacology Unit, University College London, London, UK

**Keywords:** Health care, Psychology

## Abstract

Psychedelic substances are garnering renewed interest for their potential therapeutic applications, yet the mechanisms by which challenging experiences during psychedelic use contribute to positive outcomes remains poorly understood. Here we present a mixed-methods investigation into the strategies individuals employ to navigate difficult psychedelic experiences and their relationship to emotional breakthrough. Qualitative analysis of accounts from psilocybin retreat participants (n = 16) informed the development of the Responses to Challenging Psychedelic Experiences Inventory (ReCiPE). In a subsequent online survey (n = 529), exploratory factor analysis of the ReCiPE revealed three primary response strategies: Acceptance and Reappraisal, Sensory Regulation and Physical Interaction, and Social Support and Disclosure. Exploratory correlation and multiple regression analyses demonstrated significant relationships between different types of challenges, response strategies and emotional breakthrough. Notably, Acceptance and Reappraisal, and Social Support and Disclosure strategies were positively associated with greater emotional breakthrough. Fear-related challenges were negatively associated with emotional breakthrough and involved fewer adaptive coping strategies. These findings elucidate the complex interplay between challenging experiences and adaptive responses in psychedelic contexts, offering insights for optimising therapeutic protocols and enhancing safety in both clinical and non-clinical settings.

## Introduction

Psychedelic substances, including psilocybin and N,N-dimethyltryptamine (DMT), have a long history of use in traditional healing practices and spiritual rituals^1^. Recently, there has been a resurgence of scientific interest in the therapeutic potential of these compounds, and new psychedelic compounds such as lysergic acid diethylamide (LSD), for treating various mental health conditions, such as depression, anxiety, and addiction^2–4^. While psychedelic experiences can offer profound and transformative insights^5–9^, they also pose significant challenges, often eliciting intense emotions, physical discomfort, and psychological distress^10–13^.

Despite rapid advancements in psychedelic science, the relationship between individual responses to these substances and their therapeutic outcomes remains an active area of investigation^14–18^. A growing body of evidence suggests that subjective experiences during psychedelic sessions play a crucial role in determining therapeutic efficacy^11,19–21^. Specifically, “peak” or “mystical” experiences, characterised by a sense of unity, transcendence of time and space, and deeply felt positive mood, have been consistently associated with significant improvements in well-being following psychedelic use^22–25^. Although these peak experiences have been the primary focus of research, other aspects of the psychedelic experience, including external (e.g., nature, music, preparation) and internal (e.g., understanding, mind-set, and motivation) factors, are gaining recognition as important predictors of therapeutic outcomes^18,25–32^.

Interestingly, challenging experiences—marked by intense negative emotions—have also been shown to be positively associated with therapeutic outcomes in some cases and are often perceived by users as valuable parts of their psychedelic journey^10,33–36^. These challenges supposedly provide opportunities for difficult but important insights and the resolution of personal conflicts, resulting in improved well-being and life satisfaction post-experience^28,37–39^. However, recent research has highlighted the potential for these experiences to lead to extended difficulties and setbacks in mental health^12,13,40,41^. The extent to which a challenging psychedelic experience results in either enhancement or decline in well-being is likely contingent upon the manner in which it is effectively navigated by the individual and the support provided by those with whom the experience is shared.

Recognised psychotherapeutic modalities, such as cognitive behavioural therapy (CBT), highlight the value of strategically eliciting challenging psychological states, leveraging psychological “destabilisation” to promote the learning of new adaptive responses^42–44^. This principle may be reflected in psychedelic-assisted therapy, where challenging yet manageable psychedelic experiences provide an opportunity for learning “experiential acceptance”^37^—a key component of psychological flexibility, a transtheoretical construct with broad therapeutic implications^45,46^. Importantly, the adoption of acceptance and “surrender”, rather than avoidant responses, during psychedelic experiences is thought to facilitate so-called “emotional breakthroughs”^37^. Alongside preparation^30^, “set and setting” variables^47^, and a strong therapeutic alliance^48^, effective responses and skilful navigation of challenging psychological states may therefore be particularly crucial predictors of therapeutic success.

Several gaps exist in our understanding of the strategies individuals employ to navigate challenging psychedelic experiences^11,35,49^. The absence of well-developed theories to guide clinical interventions underscores the importance of insights from exploratory research. Such research can significantly enhance our understanding of the strategies used to navigate challenging psychedelic experiences. For instance, the hypothesis that “letting go” is an optimal psychological response to intense or difficult psychedelic experiences, thereby facilitating emotional breakthroughs, remains empirically unexamined. This hypothesis warrants significant attention due to its substantial role in contemporary therapeutic protocols and user guidelines, where fostering acceptance-based responses is considered vital^46,50,51^. Furthermore, not all psychedelic challenges result in emotional breakthroughs^28^, and it remains unexplored whether different types of challenging psychedelic experiences necessitate distinct strategies for their successful resolution.

To address these knowledge gaps, we adopted a mixed-methods approach in this study, presenting two interlinked investigations. In Study 1, we used qualitative methods to analyse participants’ written accounts of their challenging psychedelic experiences, identifying key themes related to their coping strategies. These findings informed the development of the Responses to Challenging Psychedelic Experiences Inventory (ReCiPE), a novel measure designed to assess the prevalence and perceived helpfulness of specific coping strategies.

In Study 2, we administered the ReCiPE to a larger sample of individuals with prior psychedelic experience, along with measures of challenging experiences and emotional breakthrough. We conducted exploratory factor analysis to examine the underlying structure of the ReCiPE and investigated the relationships between the emergent latent factors (i.e. ReCiPe response strategies), challenging experiences, and emotional breakthroughs using correlation and regression analyses.

## Methods

All research was performed in accordance with the Declaration of Helsinki and all procedures were reviewed by, and received approval from, the University College London Research Ethics Committee (Study 1: 9437/001; Study 2: 9437/002). All participants were required to be ≥ 18 years old, proficient in English, and have prior experience with psychedelic substances. All provided electronic informed consent at the beginning of each study. In this process, participants were presented with a clear outline of the study’s purpose, procedures, their rights, and any associated risks or benefits. Consent was obtained by participants checking boxes to acknowledge their understanding and agreement before proceeding with the survey. Both studies were conducted using online surveys hosted on the Qualtrics platform. The studies were not pre-registered.

### Study 1: qualitative analysis

#### Participants and recruitment

Attendees of psilocybin retreats at four collaborating centres in the Netherlands and Mexico were recruited via email. Only attendees who had consented to be contacted for research participation were included. Additional inclusion criteria were: prior psilocybin retreat attendance and self-reported significant personal transformation coupled with distress or difficulty during ceremonies.

#### Measures

##### Demographic and contextual factors

Detailed demographic information was collected from participants, including age, gender, ethnicity, country of residence, religious denomination, and level of education. Additionally, comprehensive information about the participants’ psilocybin retreats was obtained. This included the specific retreat centre attended and the duration of their stay. Participants also reported the number of psilocybin ceremonies or dosing sessions they attended during their retreat. For each psilocybin session during their retreat, participants were asked to quantify the extent and intensity of challenging experiences. Specifically, they were asked: (1) “Approximately how much of the psychedelic experience involved challenging feelings, distress, or discomfort?” and (2) “How personally difficult or challenging was the experience?”.

##### Challenging experience narratives

The core of the survey comprised open-ended questions designed to elicit detailed narratives of participants’ challenging experiences. These questions prompted participants to describe the nature of the challenge, their responses, the resolution process, and the meaning derived from the experience. The key questions included:

1. “Please describe in detail your experience of any challenging, difficult, or distressing episodes during the psychedelic sessions (or ‘ceremonies’).”
2. “People show a variety of responses to difficult feelings and situations. How did you respond to the challenging experiences you described above?”
3. “How did the challenging experiences come to an end?”
4. “How would you describe the role that these challenging or difficult episodes played in your psychedelic journey?”

### Data analysis

#### Descriptive statistics

Descriptive statistics were calculated to summarise the demographic and contextual characteristics of the participants, as well as the extent and intensity of their challenging experiences.

#### Thematic analysis

Detailed narratives of challenging experiences, including coping strategies and resolutions, were analysed using the Braun and Clarke framework^52^. Two researchers independently coded the data using NVivo software, employing an inductive approach to ensure inter-coder reliability. Codes were iteratively refined and collated into themes following DeSantis and Ugarriza’s criteria^53^. For this analysis, coping strategies were defined as cognitive or behavioural responses employed to manage perceived difficulties during challenging psychedelic experiences. This definition aligns with cognitive-behavioural therapy concepts of adaptive or maladaptive coping. Involuntary reactions (e.g., fight-flight responses, crying) were excluded when they appeared intrinsic to the challenging experience itself, though the complexity of this distinction is acknowledged. This rigorous qualitative approach yielded key themes related to participants’ coping strategies, providing a foundation for the subsequent quantitative investigation in Study 2.

### Study 2: online survey

Study 2 aimed to build upon the qualitative findings of Study 1 by quantitatively assessing the prevalence and perceived helpfulness of coping strategies employed during challenging psychedelic experiences. The Responses to Challenging Psychedelic Experiences Inventory (ReCiPE), developed based on the themes identified in Study 1, was administered to a larger sample. This study sought to further investigate the relationships between challenging experiences, response strategies, and emotional breakthroughs using the ReCiPE scale, thereby providing a more comprehensive understanding of how individuals navigate and derive meaning from these experiences.

#### Participants and recruitment

Participants were recruited through an online survey advertised to individuals with prior psychedelic experience via targeted social media, research groups, and user forums. Inclusion criteria required participants to have had at least one experience with a classic psychedelic or analogue (psilocybin, ayahuasca, LSD, DMT, mescaline). Experiences limited to MDMA, ketamine, or other non-classic psychedelics were excluded, although combined use with classic psychedelics was permitted.

#### Measures

##### Demographic and contextual factors

Demographic information, lifetime psychedelic experience, the type of psychedelic substance consumed, and time elapsed since the experience were collected.

##### Challenging psychedelic experiences

Participants were initially asked to report whether they experienced any period of “distress, difficulty, or discomfort” during their reported psychedelic experience. Those who indicated such experiences completed the 26-item “Challenging experience questionnaire” (CEQ)^26^. The CEQ employs a 6-point Likert scale ranging from 0 = “none; not at all” to 5 = “extremely (more than ever before in my life)”. This validated measure encompasses seven factors: grief, physical distress, fear, insanity, isolation, death experiences, and paranoia. An additional item rating experiences of trauma-reliving was also included, as an exploratory item during data collection, but was not included in the analyses as this item was not part of the original validated CEQ measure.

##### Emotional breakthrough

The 6-item “Emotional breakthrough inventory” (EBI)^28^ was used to assess episodes of catharsis or emotional release following a psychedelic experience. Participants rated items on a visual analogue scale ranging from 0 (“No, not more than usual”) to 100 (“Yes, entirely or completely”). We used the EBI (rather than specific symptom scales) as our ‘outcome’ in the regression analyses for Study 2 because it is more appropriate for a mixed non-clinical participant group with varied motivations for psychedelic use.

##### Responses to challenging psychedelic experiences

To assess the prevalence and perceived helpfulness of specific coping strategies, we developed the Responses to Challenging Psychedelic Experiences Inventory (ReCiPE). The 26 items for the ReCiPE were primarily derived from the themes identified in the qualitative analysis conducted in Study 1, which aimed to identify coping strategies used by participants during challenging psychedelic experiences. These findings were supplemented by a review of the relevant literature on user experiences of challenging psychedelic states^10,11,34–36,49^. For each of the 26 ReCiPE items, participants indicated whether they had (i) not attempted the strategy, (ii) attempted a particular strategy but not found it helpful, (iii) attempted it and found it somewhat helpful, and (iv) attempted it and found it substantially helpful. Note that this response scheme was intended primarily to be descriptive (frequencies of the four response types). Because the descriptive labels for the four response options were neither symmetrical around an intuitive midpoint nor strictly ordinal, the scoring scheme was not suitable for standard psychometric evaluation techniques. Instead, to preliminarily examine its factor structure, ReCiPE responses were transformed into a binary scoring scheme (0 = did not try; tried, was not helpful; 1 = tried and somewhat/substantially helpful) and appropriate estimation methods were then used to evaluate its dimensionality. ReCiPE ‘scores’, e.g. as used in the regression analyses, therefore refer to the number of strategies judged to be helpful (total, and per subscale). The full scale is available in Supplementary Material S.II.

### Data analysis

Statistical analyses were conducted using Mplus (version 8.7; Muthén & Muthén, 2022), Python (version 3.11.0; Python Software Foundation, 2022), and JASP (version 0.14.1; JASP Team, 2024). A significance threshold of α = 0.05 was used.

#### Descriptive statistics

Descriptive statistics for demographic variables, psychedelic experience characteristics, and the prevalence of challenging experiences and responses are presented as means (± SD), medians, modal values with ranges, or counts/frequencies (and percentages).

#### Factor analysis

The binary scoring of the ReCiPE scale facilitated dimension reduction and the identification of underlying factors through exploratory factor analysis (EFA). EFA was conducted testing two, three, and four-factor solutions. We employed robust weighted least squares adjusted estimation (WLSMV) applied to a tetrachoric correlation matrix to ensure robust results given the binary nature of the data. Oblique (Geomin) rotation was used to allow the factors to covary, reflecting the likelihood that coping strategies might be interrelated.

To determine the optimal number of factors to retain, we used several criteria: model fit statistics (RMSEA, comparative fit and Tucker-Lewis indices with values < 0.05 and > 0.9 respectively), scree plots, theoretical considerations and interpretability (e.g. selecting the solution with the fewest items that cross-loaded on > 1 factor and had factor loadings < 0.4). Theoretical considerations in particular ensured that the factors were meaningful and interpretable within the context of coping strategies for challenging psychedelic experiences.

#### Reliability

Internal consistency of the CEQ (α ≥ 0.928) was excellent. Although the EBI includes a series of distinct questions, the 6-item version of the EBI used here loaded on a single factor (accounting for 71.4% of variance) and also had high internal consistency (α = 0.918), justifying the use of a single average score in the regression analyses (below). Kuder-Richardson’s Formula 20 (‘KR-20’) for assessing the internal consistency of binary response variables (relevant for the ReCiPE;^54^) gave values that were indistinguishable from Cronbach’s alpha. Therefore, no special adjustments to Cronbach’s formula were used to determine the internal consistency of the ReCiPE and its subscales (reported below).

#### Correlations

Pearson’s correlation coefficients were calculated to assess the relationships between challenging psychedelic experiences (as measured by the CEQ factors: Fear, Grief, Physical Distress, Insanity, Isolation, Death, and Paranoia), response strategies (as measured by the ReCiPE factors: *Acceptance and Reappraisal*, *Sensory Regulation and Physical Interaction*, and *Social Support and Disclosure*), and emotional breakthrough (as measured by the Emotional Breakthrough Inventory average).

#### Multiple regression

A multiple linear regression analysis was conducted to investigate the predictive power of challenging psychedelic experiences and response strategies on emotional breakthrough. Variables that demonstrated significant correlations with emotional breakthrough in the previous analysis were selected as predictors for the regression model. The assumptions of multiple linear regression, including linearity, normality, homoscedasticity, and independence of residuals, were thoroughly assessed using residual plots, Q-Q plots, and the Breusch-Pagan test. The Durbin-Watson statistic was employed to evaluate the independence of residuals, while Tolerance and VIF values were used to assess multicollinearity among the predictors. The overall significance of the regression model was determined using an F-test, and the contribution of each predictor was assessed using t-tests. The adjusted R^2^ was reported to indicate the proportion of variance in emotional breakthrough explained by the model, and the R^2^ change and its significance (F change and p-value) were also reported to demonstrate the additional variance explained by the predictors compared to the intercept-only model. Unstandardized coefficients (B), standardised coefficients (β), and their 95% confidence intervals were reported to provide a comprehensive understanding of the relationships between the predictors and the dependent variable.

## Results

### Study 1: qualitative analysis

#### Participant characteristics

Sixteen participants (n = 8 from the Netherlands, n = 8 from Mexico) completed the survey. They attended retreats lasting 3–7 days, participating in up to 3 psychedelic ceremonies. The median time since attendance was 6 months. Participants’ mean age was 45 years, with 9 identifying as female and 7 as male. Most participants (n = 13) reported no religious affiliation, and nearly all (n = 15) had completed graduate or postgraduate education. Further demographic details are presented in Supplementary Material S.I.

#### Thematic analysis

Participants reported various challenges during psychedelic ceremonies, including the emergence of past traumas, prolonged anxiety or panic, intense grief, physical distress, and feelings of dissatisfaction with the experiences. Despite the distressing nature of these experiences, participants noted several positive outcomes, such as improvements in personal relationships, increased honesty, and a heightened capacity for forgiveness.

Our thematic analysis of participant accounts identified four main themes related to participants’ responses and coping strategies during challenging psychedelic experiences: *Inner Responses* (e.g., introspective strategies such as accepting and observing the experience, engaging in self-talk and reassurance, interrogating and commanding the challenge, and making meaning of the experience), *Embodied Practice and Engagement with the Environment* (e.g., intentional breathing, physical movement, sensory engagement with the environment, and seeking sleep), *Interpersonal Responses* (e.g., avoiding social interactions, seeking help, and disclosing personal experiences as part of their coping strategies), and *Facilitator Responses* (e.g., the critical role of facilitators in providing physical touch, reassurance, and introducing new elements to assist participants during challenging moments). Table 1 summarises these themes, sub-themes, and illustrative quotes. For a more comprehensive presentation of the qualitative findings, please refer to Supplementary Material S.II, S.III and S.IV.

**Table 1:** Summary of themes, sub-themes, and illustrative quotes from Study 1.

Theme	Sub-themes	Illustrative quote(s)
1. Inner responses	1.1 Accepting and observing	“…Meditating earlier helped where I learnt to observe thoughts and not fight them. This allowed me to smoothly go further in the journey and view the issue from different angles” (P14) “My physical pain lessened when I accepted it as part of my learning.” (P8) “I tried to embrace it—lean into it.” (P2)
	1.2 Self-talk, re-assurance and “reminders to self”	“I gave my last resolution to myself of “I can do this”” (P3) “To feel like you’re being closed on and there is nothing you can do is terrifying, especially because you don’t know when it will end. But then I would help myself by remembering to trust the plant medicine and go into the experience. It was there for a reason.” (P3)
	1.3 Interrogating and commanding the challenge	“When I saw the giant spider, I asked it two questions, ‘what are you trying to tell me’? ‘Are you medicine’? I didn’t get any type of response or an answer to either question. So, I decided to take matters into my own hands and said, ‘if you’re not medicine you must leave now’. And immediately the spider dissolved into what looked like a small pile of powder on the ground.” (P5) “The anxiety and fear stopped abruptly when I took my personal responsibility and faced it and in a figurative sense told it to go away. I took my own aggressiveness, which I normally hardly ever feel, as a source to help myself.” (P8)
	1.4 Interpretation and meaning making	“At that point I literally felt my heart break. I thought I was having a heart attack, but I remembered the discussion on sacred surgery and knew this was symbolic. After that I felt extreme sadness and grief.” (P2) “I realised that in all my cells and body parts all the suffering, pain, sorrow is stored. Not only of mine, but from all human beings of history and all their lives.” (P8)
	1.5 Journaling	“The following 3 h were spent feverishly journaling while in hysterics. Crying, laughing, more crying, more laughing. […] I thought of my family and friends and all of the relationships in my life that may have been impacted by this.” (P10) “Feeling of uncertainty. Then something had told me to bring my journal and it was the first time to do so [during the] ceremony. Then I knew what I had to do which caused more sobbing. Forgiveness. I had to write. I wrote: I forgive you, I forgive you, I forgive you, I forgive you for breaking my heart and not knowing why” (P12)
2. Embodied practice and engagement with the environment	2.1 Intentional use of breath	“I remembered what one of the guides said during the preparation: to breathe. I focused on my breathing, and it helped me stay grounded and feel some safety.” (P15) “I definitely used square breathwork, and at least during one experience I connected with the actual sound of the waves which were at our property, and used them as a way to work through the labour” (P3)
	2.2 Opening the eyes and moving around (v.s. closing the eyes and remaining still)	“Soon I realised I needed to move or sway to try to help with the nausea. We were encouraged to try to remain laying with eye masks on to let the inner self work, so I felt bad about disobeying the guidelines” (P3) “A while after the second dose was taken, I decided to move around a bit. I had felt confined to the cot for a long time, so I decided to sit up and look around. I gave up on the hope that I would have a true psychedelic experience, and leaned into the beauty of the ceremony” (P6)
	2.3 Sensory engagement with the environment and participation in the ceremony	“When I got up and started moving around, I was able to take in all of the beauty of the ceremony. This helped me take my mind off of my disappointment […] Dancing along on the edge of the ceremony space was the most impactful.” (P6) “Going outside, feeling the cold earth beneath my bare feet helped, as did stoking the fire in the pit outside.” (P11) “[…] every time I felt like I was getting somewhere, an external distraction would pull me out of the moment again and again. It got so frustrating that I finally pulled the blindfold off and spent the rest of the trip enjoying the sounds of the jungle as I gazed at the stars above” (P10)
	2.4 Leaving the ceremony	“I had been told to lean into the difficult times—that it would end. But it didn’t. I wanted to get the medicine out of my system. It was unbearable. Towards the end when others were dancing—I just needed to get outside.” (P2) “I suddenly felt as if […] I was witnessing my own death. This caused a lot of panic. I thought to myself that I had to do something “normal” to convince myself I was alive. I went to the toilet, sat there, nothing happened” (P11)
	2.5 Sleep	“I was anxious and remember a participant said, ‘you will need this to just marinate so there is nothing that can be done now. Try to sleep.’” (P13) “I finally fell asleep around 3 AM and when I woke up the next day, I felt normal again.” (P4)
3. Interpersonal responses	3.1 Avoiding social interactions	“I tried to avoid others in the group because I couldn’t summon the proper emotional responses to what they were saying, and I didn’t want to hurt anyone’s feelings, so I went to my room” (P4) “When the ceremony was done, I couldn’t get out of the space quick enough. I didn’t want to be with anyone” (P12)
	3.2 Asking for help	“I had a full anxiety attack and asked for help and was held and helped with my breathing” (P12) “[…] I found myself and my body in this lost state of fear with no way out. I stayed here WAY too long and somehow found it in me to rip my eye mask of and ask a facilitator for a hand” (P16) “Although this was the toughest part, it was probably the most healing. I learned a lot from this experience—I learned to ask for help” (P16)
	3.3 Disclosure and “confession”	“Would I tell the outside world about this? Would I tell my wife? Would this mean my marriage was going to fail? I started confronting these questions in hysterical tears by myself, then realised that the only way to truly confront this was to speak it out loud. So, I raised my hand and called on one of the facilitators.” (P10) “When I finally told the story, I felt a weight leave my body like I’ve never felt before. Tears of sadness turned to tears of joy, but still, lots of tears!” (P10) “At that point, another facilitator came over with the only other person who had given birth at the retreat, and we two mothers embraced. We cried and apologised to each other, both as mother and daughter. We forgave each other for how we are doing our best, but we are still human and imperfect.” (P3)
4. Facilitator Responses	4.1 Physical touch and reminders to breathe	“It came to an end when a facilitator came over and held my hand, stroked my head, and helped me to breathe. I was then able to find my regular breathing again and navigate my way out of a dark, dark place” (P16)
	4.2 Listening	“It took me 30 min to speak a single word, but she held my hand and waited until I was ready. She “held space” for me as they like to say.” (P10) “Towards the end when others were dancing—I just needed to get outside. Thankfully someone sat with me and just listened and talked although I felt like a failure that I had to do that.” (P2)
	4.3 Reassurance	“I tried to grasp reality by asking a guide what day it was and what the time was but he gave a very ‘you’re in this moment/you’re right where you need to be’ answer which really didn’t help.” (P11) “Internally my mind was racing and I remember sweating profusely. I remember being reassured by the lead guide who reminded me that I’d had a large amount of truffles and was safe and had done really well. That was incredibly reassuring.” (P11)
	4.4 Rhythm and sound	“Fortunately, the lead facilitator recognized it was time for doula-like help. She came over and started humming in beat with the instruments that were being played by other facilitators. I started humming with her and we put our throats against each other. It was through that physical, audible, and vibrational connection that I found the power to give the final ecstatically painful push.” (P3) “The music they set our trip to played a huge part as well in ensuring the trip was beneficial, even in the worst of times.” (P10)
	4.5 Introducing new elements	“After hours of feeling very little, other than frustration that the ‘mushrooms didn’t love me either’—one of the sitters came and handed me a rose quartz crystal. At that point I literally felt my heart break.” (P2)

These findings informed the development of the Responses to Challenging Psychedelic Experiences Inventory (ReCiPE) used in Study 2, which aimed to quantitatively assess the prevalence and perceived helpfulness of specific coping strategies identified in the qualitative analysis. The ReCiPE can be found in Supplementary Material S.V.

### Study 2: online survey

#### Descriptive statistics

Of the 869 individuals who completed the survey, 555 (64.24%) reported having experienced some form of challenge during their psychedelic experience, of whom 529 (95.31%) provided data for the subsequent analyses. The sample was balanced in terms of gender, with 243 (45.94%) identifying as male, 256 (48.39%) as female, and 30 (5.67%) as non-binary or ‘other’. The mean age of the sample was 37.71 years (SD = 10.88). Detailed demographic and sample characteristics are presented in Supplementary Material S.VI.

**Fig. 1:**
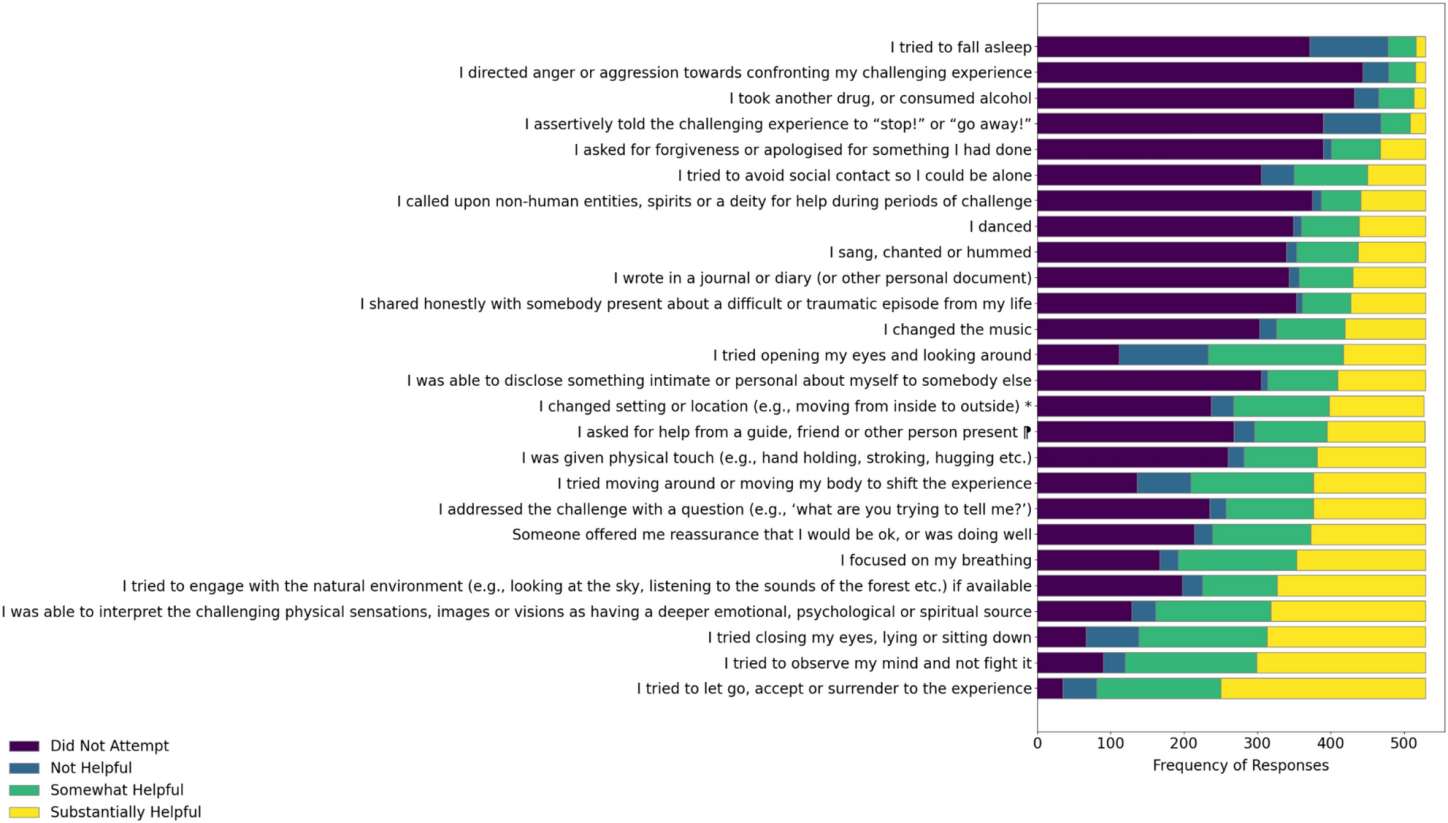
Frequency of responses for each ReCiPE item (n = 529). The horizontal stacked bar chart shows the frequency of responses for each ReCiPE item, indicating how many participants rated each strategy as “Did Not Try”, Not Helpful”, “Somewhat Helpful” or Substantially Helpful”.

Supplementary Material S.VII presents the frequencies with which each strategy was either not tried, tried and found not to be helpful, or tried and found to be somewhat or substantially helpful. As shown in Fig. 1, the strategies most commonly employed and found to be helpful (combining somewhat helpful and substantially helpful scores) were “I tried to let go, accept or surrender to the experience” (84.5% of participants), “I tried to observe my mind and not fight it” (77.3%) reporting helpfulness, and “I tried closing my eyes, lying or sitting down” (73.9%). The least commonly helpful strategies were “I directed anger or aggression towards confronting my challenging experience” (9.4%), “I tried to fall asleep” (9.7%), “I assertively told the challenging experience to ‘stop!’ or ‘go away!’” (10.6%), and “I took another drug, or consumed alcohol” (12.1%).

In addition to summarising ReCiPE responses at the per-item level, we also evaluated the number of strategies each individual employed and found (somewhat/substantially) helpful. Summing across the number of strategies per participant showed that, on average, they used 11.25 ± 4.78 strategies that were judged to be helpful during their challenge (mode = 10).

#### Factor analysis

The 26 items with transformed binary scoring (see Methods) appeared to be best represented by three factors (Table 2). The two-factor model had poor fit (RMSEA > 0.05; TLI and CFI < 0.9) and although the three- and four-factor models showed similar fit indices (RMSEA < 0.05; TLI and CFI > 0.9), the items appeared to coalesce in a more theoretically coherent way in the three-factor model, which also had fewer cross-loaded items (Table 2). Thematically, items within the factors were related to: *Acceptance and Reappraisal* (Factor 1), *Sensory Regulation and Physical Interaction* (Factor 2) and *Social Support and Disclosure* (Factor 3). Factors 1 and 2 were modestly correlated (r = 0.330), although the other two pairwise associations were weak suggesting that overall, the factors are tapping relatively distinct constructs.

**Table 2:** Geomin rotated loadings for the ReCiPE scale.

Item	Description	Factor 1	Factor 2	Factor 3
1	I tried to let go, accept or surrender to the experience	**0.60**	0.26	0.03
2	I tried to observe my mind and not fight it	**0.72**	0.23	−0.06
3	I addressed the challenge with a question (e.g., ‘what are you trying to tell me?’)	**0.71**	0.04	−0.04
4	I assertively told the challenging experience to “stop!” or “go away!”	**(0.17)**	0.15	−0.14
5	I directed anger or aggression towards confronting my challenging experience	**0.43**	−0.01	0.04
6	I was able to interpret the challenging physical sensations, images or visions as having a deeper emotional, psychological or spiritual source	**0.66**	0.19	−0.01
7	I wrote in a journal or diary (or other personal document)	**0.56**	−0.10	0.05
8	I focused on my breathing	**0.52**	0.15	0.04
9	I tried closing my eyes, lying or sitting down	**0.42**	0.31	0.03
10	I tried moving around or moving my body to shift the experience	0.01	**0.76**	−0.06
11	I tried opening my eyes and looking around	0.03	**0.73**	−0.06
12	I danced	0.06	**0.63**	−0.02
13	I sang, chanted or hummed	0.36	**0.44**	0.02
14	I changed the music	**−**0.01	**0.58**	−0.01
15	I changed setting or location (e.g., moving from inside to outside)	**−**0.37	**0.85**	0.04
16	I tried to engage with the natural environment (e.g., looking at the sky, listening to the sounds of the forest etc.) if available	–0.22	**0.82**	–0.11
17	I tried to fall asleep	0.02	**(0.27)**	–0.11
18	I tried to avoid social contact so I could be alone	0.17	**0.42**	–0.28
19	I asked for help from a guide, friend or other person present	0.03	0.02	**0.75**
20	I was able to disclose something intimate or personal about myself to somebody else	0.34	0.03	**0.75**
21	I shared honestly with somebody present about a difficult or traumatic episode from my life	0.43	–0.04	**0.78**
22	I asked for forgiveness or apologised for something I had done	**0.56**	0.01	0.22
23	I was given physical touch (e.g., hand holding, stroking, hugging etc.)	0.02	0.15	**0.77**
24	Someone offered me reassurance that I would be ok, or was doing well	–0.06	0.16	**0.80**
25	I called upon non-human entities, spirits or a deity for help during periods of challenge	**0.56**	–0.03	0.11
26	I took another drug, or consumed alcohol	–0.23	**(0.37)**	0.06
Correlations				
Factor 1		–		
Factor 2		0.330*	–	
Factor 3		0.118	0.217	–

*p < 0.05. Items in bold within a column are treated as belonging to the same factor; items in brackets had factor loadings < 0.4.

Items that did not load strongly on any of the three factors (item 4: ‘I assertively told the challenging experience to “stop!”, or “go away!”’; item 17: ‘I tried to fall asleep’ and item 26: ‘I took another drug, or consumed alcohol’) were among the less commonly employed strategies. Some items showed cross-loadings (items 9 and 13).

#### Reliability

The internal consistency of the full ReCiPE was α = 0.801. Removal of the items with low factor loadings (items, 4, 17, 26) had virtually no effect on reliability (α = 0.806). Similarly, inclusion/exclusion of the weakly loading items within individual factors had little impact on their internal consistencies: α = 0.726 for Factor 1 (if item 4 included: α = 0.718), α = 0.731 for Factor 2 (if items 17 and 26 included: α = 0.716), and α = 0.786 for Factor 3. On balance, given the preliminary nature of the factor analysis, we did not remove any items from the scale and as such, Factor 1 consisted of 11 potential strategies, Factor 2, 10 strategies and Factor 3, five strategies.

Based on the factor structure outlined in Table 2, the average (and modal) number of strategies used and found to be helpful for each of the factors was: Factor 1: 5.25 ± 2.39 (mode = 5 ; possible range = 0–11), Factor 2: 3.82 ± 2.41 (mode = 4; possible range = 0–10), Factor 3: 2.17 ± 1.80 (mode = 0; possible range = 0–5).

#### Correlations

Pearson’s correlation coefficients (Table 3) revealed significant associations between several challenging psychedelic experiences, response strategies, and emotional breakthroughs. Fear, Grief, Physical Distress, and Death were significantly correlated with EBI average, while Insanity, Isolation, and Paranoia were not. All three response strategies (*Acceptance and Reappraisal*, *Sensory Regulation and Physical Interaction*, and *Social Support and Disclosure*) showed significant correlations with EBI average. Given the binary scoring system used for the ReCiPE, higher scores on its subscales imply the use of a greater number of strategies perceived to be helpful. As such, the positive association between Grief and Acceptance/Reappraisal (r = 0.19, p < 0.001) implies that Grief-related challenges were associated with the deployment of a larger number of helpful acceptance/reappraisal strategies. Conversely Fear was associated with the use of fewer helpful acceptance/reappraisal strategies.

**Table 3:** Pearson’s correlations between types of challenging psychedelic experiences (CEQ subscales), response strategies (recipe subscales), and emotional breakthrough.

	Variable	1	2	3	4	5	6	7	8	9	10	11
CEQ	1. Fear	–										
	2. Grief	0.30***	–									
	3. Physical distress	0.51***	0.35***	–								
	4. Insanity	0.68***	0.24***	0.44***	–							
	5. Isolation	0.56***	0.51***	0.39***	0.47***	–						
	6. Death	0.39***	0.22***	0.37***	0.41***	0.26***	–					
	7. Paranoia	0.38***	0.21***	0.35***	0.45***	0.43***	0.21***	–				
ReCiPE	8. Acceptance and reappraisal	−0.20***	0.19***	0.11*	−0.18***	−0.07	0.15***	− 0.13**	–			
	9. Sensory regulation and physical interaction	−0.14**	0.04	0.07	−0.12**	0.04	−0.09*	0.05	0.32***	–		
	10. Social support and disclosure	0.02	0.24***	0.11**	−0.04	0.06	0.08	0.05	0.30***	0.19***	–	
11. EBI		−0.14**	0.41***	0.10*	−0.07	−0.03	0.20***	−0.08	0.50***	0.18***	0.27***	–

* p < .05, ** p < .01, *** p < .001.

#### Multiple regression

The multiple linear regression model with fear, grief, physical distress, death (CEQ factors) and acceptance/reappraisal, sensory regulation/physical interaction and social support/disclosure as predictors (Table 4) significantly predicted emotional breakthrough (F(7, 519) = 50.93, p < 0.001), explaining 40% of its variance (adjusted R^2^ = 0.40). The assumption checks revealed that the linearity, normality, and homoscedasticity assumptions were satisfactorily met. The Durbin-Watson statistic (1.96) indicated no significant autocorrelation in the residuals, and the Tolerance (range: 0.59–0.87) and VIF (range: 1.15–1.70) values were within acceptable limits, suggesting no multicollinearity among the predictors.

**Table 4:** Multiple regression analysis predicting emotional breakthrough (EBI average).

Predictor		β	SE	t	p
(Intercept)		29.33	3.30	8.89	** < .001**
CEQ factors	Fear	−0.24	0.81	−5.50	** < .001**
	Grief	0.37	0.72	9.78	** < .001**
	Physical distress	−0.02	0.93	−0.40	0.693
	Death	0.17	0.59	4.45	** < .001**
ReCiPE factors	Acceptance and reappraisal	0.33	0.50	8.29	** < .001**
	Sensory regulation and physical interaction	0.02	0.45	0.63	0.527
	Social support and disclosure	0.07	0.60	2.05	**0.041**

F(7, 519) = 50.932, p < .001, Adjusted R^2^ = 0.399, ΔR^2^ = 0.407, ΔF = 50.932, p < .001.

Several variables emerged as significant predictors of emotional breakthrough. Fear (β = −0.24, 95% CI [−0.30, −0.14], t = −5.50, p < 0.001) and Grief (β = 0.37, 95% CI [0.28, 0.42], t = 9.78, p < 0.001) from the challenging experience questionnaire (CEQ) were found to be significant predictors, with Grief positively associated with emotional breakthrough and Fear negatively associated. The Death factor from the CEQ also significantly predicted emotional breakthrough (β = 0.17, 95% CI [0.07, 0.19], t = 4.45, p < 0.001). Among the response strategy factors of the ReCiPE, *Acceptance and Reappraisal* (β = 0.33, 95% CI [0.16, 0.26], t = 8.29, p < 0.001) and *Social Support and Disclosure* (β = 0.07, 95% CI [0.00, 0.12], t = 2.05, p = 0.041) were significant positive predictors of emotional breakthrough.

The unstandardized coefficients (B) provide insight into the change in emotional breakthrough for each unit change in the predictor variables. For instance, a one-unit increase in Grief is associated with a 0.37-unit increase in emotional breakthrough, holding all other predictors constant. Conversely, a one-unit increase in Fear is associated with a 0.24-unit decrease in emotional breakthrough, holding all other predictors constant. These findings highlight the complex relationships between challenging psychedelic experiences, response strategies, and emotional breakthrough, with both positive and negative associations observed.

## Discussion

This research examined the effectiveness of strategies used by individuals to navigate challenging psychedelic experiences. In Study 1, we conducted a qualitative analysis of participant accounts to identify key response strategies, which informed the development of the Responses to Challenging Psychedelic Experiences Inventory (ReCiPE). This novel measure was designed to assess the prevalence and perceived helpfulness of specific response strategies. In Study 2, we explored the factor structure of the ReCiPE and examined the relationships between its factors, challenging experiences and emotional breakthroughs, providing a preliminary understanding of how individuals manage and derive meaning from challenging psychedelic experiences.

Study 1 provided insights into the diverse strategies individuals use to navigate challenging psychedelic experiences. Through thematic analysis of written reports, we identified four main themes, reflecting a spectrum of cognitive, behavioural, and social response strategies. The Inner Responses theme included introspective strategies such as acceptance, self-talk, interrogating the challenge, and meaning-making. The Embodied Practice and Engagement with the Environment theme emphasised physical strategies like intentional breathing, movement, and sensory engagement to manage difficult experiences. The Interpersonal Responses theme highlighted social dynamics, where participants either avoided social interactions, or alternatively sought help, or disclosed personal experiences. Lastly, the Facilitator Responses theme underscored the critical role of guides or therapists in providing physical and emotional support and introducing new elements to assist participants during challenging moments. This aligns with existing evidence that the quality of this relationship predicts emotional breakthrough and improvements in mental health^48^. Examination of responses and thematic analysis led to the development of the 26-item ReCiPE scale (Table 2, Fig. 1).

Study 2 aimed to assess the prevalence and perceived effectiveness of specific coping strategies, and to quantitatively examine the relationships between challenging psychedelic experiences, types of response strategies (ReCiPE subscales), and emotional breakthroughs. The exploratory factor analysis of the ReCiPE revealed a three-factor structure: Acceptance and Reappraisal, Sensory Regulation and Physical Interaction, and Social Support and Disclosure. These factors are closely aligned with the themes identified in Study 1, providing preliminary support for the ReCiPE’s validity in capturing key dimensions of individuals’ responses to psychedelic challenges. The analysis also showed that higher scores on all of the ReCIPE subscales were correlated with emotional breakthrough, confirming that emotional breakthrough during challenging experiences involve participants adopting a higher number of helpful coping strategies.

Descriptive analyses revealed that strategies fostering acceptance and cognitive observation were frequently used and perceived as most effective in managing challenging psychedelic experiences. The multiple regression suggested that strategies involving Acceptance and Reappraisal, and Social Support and Disclosure may be particularly associated with experiences of emotional breakthrough. However, some of the less frequently used strategies were also found to be substantially helpful by some individuals, suggesting a one-size-fits-all approach to managing psychedelic challenges may be inadequate. Practically, although certain strategies may in general be more effective, therapists should be well-versed in a broad spectrum of response strategies and recognise that different strategies may be more or less effective for different individuals, or specific types of challenges. Future research should systematically evaluate the efficacy of diverse response strategies across various individual differences and contexts and assess the role of specific psychological traits in strategy effectiveness. These insights would inform evidence-based protocols for managing challenging psychedelic experiences, enhancing therapeutic outcomes and safety in clinical applications.

The correlations among the three ReCIPE factors, the challenging experiences (CEQ) subscales, and emotional breakthrough (EBI) revealed intricate relationships, which were also further tested in the multiple regression model. Firstly, the analysis suggests that experiences of emotional breakthrough during psychedelic use are more likely to occur alongside certain kinds of challenging experiences rather than others. In particular, heightened fear (including experiences of panic and anxiety) was less likely to co-occur with emotional breakthrough in our sample, whilst grief-related challenges (which include experiences of sadness and despair) and death experiences (involving the profound experience of one’s own death or the feeling of dying^26^) were more commonly associated with emotional breakthrough. Further study would be required to confirm this association and investigate causal pathways. It may be that participants in our sample who reported heightened fear-based challenges became stuck in prolonged negative feedback loops during their psychedelic use, thus inhibiting experiences of emotional breakthrough^37^. Conversely, heightened sadness and death type experiences, although challenging, may have been experienced alongside profound insights or processing of loss^55^.

Notably, there were also varying associations between the subscales on the ReCIPE and different kinds of psychedelic challenge. These associations raise the question as to whether different kinds of coping strategies may elicit or inhibit different kinds of psychedelic challenge, and in turn whether different kinds of challenges tend to elicit and reward different kinds of coping. It may be for example that people who are able to successfully adopt Acceptance and Reappraisal and Social Support and Disclosure strategies are better able to manage or tolerate anxiety during psychedelic use, which allows for the emergence of profound grief or death experiences, and thus more emotional breakthrough. Indeed, fear is known to induce avoidance behaviours^56^, which can interfere with acceptance-based strategies commonly used in therapeutic settings^57–60^. Fear-induced impairment in adaptive cognitive-affective strategies may lead to cognitive and emotional disengagement from challenging psychedelic content, potentially disrupting the integration of new insights and emotional processing crucial for therapeutic progress.

In contrast, grief experiences may prompt individuals to engage in adaptive cognitive processes, such as acknowledging emotions without suppression and reinterpreting loss and sadness in a new context, aligning with research on the importance of acceptance and cognitive reappraisal in grief processing^61–71^. Furthermore, grief experiences may increase openness to seeking and receiving social support, which is beneficial in grief management^72–74^. Similarly, individuals encountering death-related experiences during psychedelic experiences may be more likely to engage in cognitive processes that involve accepting and reinterpreting these existential challenges. Acceptance-related responses in particular align with the concept of 'surrender,' which has been identified as a crucial factor in determining the emotional valence of ego-death experiences^75–78^. These hypotheses would require further investigation. The above associations could also be better explained by differences in set (for example anticipatory anxiety, or personality variables) and setting (for example recreational as opposed to therapeutic settings) that make different kinds of challenges and coping strategies more or less salient.

A better understanding of the above associations and explanatory mechanisms could improve clinical efficacy and safety in the therapeutic use of psychedelics. Indeed, our findings suggest that fear management during the acute psychedelic experience may be an important determinant of outcome in psychedelic-assisted therapies. Acceptance and Commitment Therapy (ACT), which promotes psychological flexibility through acceptance and mindfulness, provides a promising framework for addressing this issue^79^. By encouraging individuals to embrace their internal experiences without attempting to alter them, ACT may help to manage fear more effectively in psychedelic contexts^50,80–83^. Integrating ACT-based training into psychedelic therapy preparation by equipping individuals with skills in mindfulness, acceptance, cognitive defusion, and values-based action may decrease the negative direct effect of fear on emotional breakthroughs and increase the positive indirect effect through improved acceptance and reappraisal strategies. Additionally, cognitive reappraisal techniques, which involve reinterpreting fear-inducing situations to reduce stress responses, may support emotional breakthroughs by fostering a more adaptive perspective during intense experiences^84,85^. Incorporating these evidence-based strategies into psychedelic therapy protocols may significantly improve therapeutic outcomes by mitigating the detrimental effects of fear and promoting adaptive coping mechanisms. Their inclusion in preparation for the psychedelic experiences is especially important given that emotional regulation strategies vary considerably between individuals^86^, and those with a habitual tendency to suppress or avoid intense emotions may require additional support in developing adaptive, though effortful, coping strategies during challenges. This may be the case particularly in clinical populations, including people with severe mental health presentations, who may or may not be suitable for psychedelic therapy.

While this study provides valuable insights into strategies for navigating challenging psychedelic experiences, several limitations should be acknowledged. First, the self-selected nature of both the qualitative and quantitative samples may have introduced selection bias, limiting the generalisability of the findings to broader populations and clinical contexts. The participant pool primarily consisted of experienced psychedelic users from Western cultures, potentially skewing the results towards individuals who have developed effective coping strategies over time and limiting the cross-cultural applicability of the findings. Participants were also self-reporting retrospectively on experiences that occurred in some cases several years prior, raising questions about the reliability of their reports. The sample included in Study 1 was also recruited on the basis of reporting significant personal transformation consequent to their psychedelic ceremonies, and most participants described beneficial outcomes.sect A different set of responses and strategies might be found in a sample reporting mostly no significant change, or negative outcomes. Moreover, the setting and motivations for psychedelic use in sample Study 2 were likely diverse and may diverge from those seeking psychedelic assistant therapy for psychiatric conditions. Future research should employ more rigorous sampling techniques to obtain diverse and representative samples, including first-time users and clinical populations, and investigate these phenomena across different cultural contexts to enhance the generalisability and relevance of the findings.

Second, although the ReCiPE is based on qualitative findings and existing literature, it is a novel measure that requires further validation. The original scoring system we devised was primarily designed to provide a descriptive account of the prevalence and efficacy (helpfulness) of the various coping responses. The necessary transformation of the original asymmetric scoring to a binary scoring system used in the EFA and exploratory qualitative analyses resulted in a loss of information and introduced some challenges in interpretation. For example, the ReCiPE total/subscale scores derived from the binary scoring relate to the number of strategies perceived to be helpful rather than degree of ‘helpfulness’ per se. We recommend further validation of the ReCiPE using an alternative (interval) scoring scheme to enhance its utility in research on challenging psychedelic experiences.

Lastly, the clinical implications of the findings warrant further investigation. While the study provides a valuable foundation for understanding the complex dynamics of challenging psychedelic experiences and the strategies used to navigate them, its direct applicability to clinical contexts may be limited. The set and setting of psychedelic experiences in therapeutic environments can differ significantly from those in recreational or retreat settings, potentially influencing the nature of challenges encountered and the effectiveness of various coping strategies. Future research should investigate these phenomena in controlled clinical settings, using rigorous methodologies and validated measures, to inform the development of targeted interventions and optimise therapeutic outcomes in psychedelic-assisted therapies. Whilst this study explored associations between challenging experiences and emotional breakthrough, a multitude of other relevant outcomes could also be studies, including wellbeing measures or measures of psychological insight^27^. It should also be noted that our discussion is neutral on whether the intentional activation of challenging states during psychedelic use is recommended or therapeutic. Current models of psychedelic action suggest that an open and detached stance to what arises in the psychedelic state is preferable, which may or may not include challenges^37^.

In summary, our findings suggest that the paradoxical positive therapeutic role of challenging psychedelic experiences may depend on the kind of challenge faced and the individual’s ability to employ adaptive response strategies. The ReCiPE factors of Acceptance and Reappraisal and Social Support and Disclosure highlight the importance of integrating internal cognitive processes and interpersonal support into psychedelic therapy protocols. Acceptance and Reappraisal strategies, aligning with approaches like ACT and Mindfulness Based Stress Reduction (MBSR)^87,88^, showed a positive association with emotional breakthroughs, suggesting the value of incorporating mindfulness and acceptance-based practices into psychedelic interventions^50,80,83,89–91^. Social Support and Disclosure strategies also showed positive associations with emotional breakthroughs, aligning with recent research on interpersonal support in psychedelic contexts^48,92,93^. These patterns emphasise the complex interplay between emotional experiences and coping mechanisms, highlighting the need for flexible, tailored therapeutic approaches. The ReCiPE’s development and preliminary analysis offer a valuable tool for future research, while the negative association between fear and emotional breakthroughs warrants particular attention in clinical protocols. As psychedelic science progresses, future research should address this study’s limitations by using longitudinal designs, conducting controlled clinical trials, and recruiting diverse samples to strengthen the evidence base and clarify the causal mechanisms underlying psychedelics’ therapeutic effects.

## Supplementary Information

Supplementary Information.

## Data Availability

Anonymised datasets generated and analysed during the current study are freely available from the corresponding author on reasonable request. The qualitative data used in Study 1 however contains potentially identifying information, and so cannot be shared, although the reader is signposted to the supplementary information for more detail on the nature of the challenges and outcomes reported by the sample.
