# Supplementary material for: Strategies for resolving challenging psychedelic experiences: insights from a mixed-methods study
Source: Sci Rep. 2024 Nov 21;14:28817. doi: 10.1038/s41598-024-79931-w (PMC11582610; doi:10.1038/s41598-024-79931-w)
Supplement: Supplementary file 1 — Supplementary Information. [file 41598_2024_79931_MOESM1_ESM.docx]

# Supplementary Material: ‘Strategies for Resolving Challenging Psychedelic Experiences: Insights from a Mixed-Methods Study’

Authors: Maximillian Wood, Rosalind G. McAlpine and Sunjeev K. Kamboj

# S.I Study 1 Participant Characteristics (N=16)

| **Category (N=16)** | **M** | **SD** | **Range** |
| --- | --- | --- | --- |
| Age | 45.21 | 11.65 | 28-62 |
| Days Spent at Retreat | 5.07 | 1.33 | 3-7 |
| Number of Ceremonies | 2.07 | 0.70 | 1-3 |
|  | **Subcategory** | **N** | **%** |
| Gender | Male | 7 | 43.75 |
|  | Female | 9 | 56.25 |
| Ethnicity | White/Caucasian | 10 | 62.5 |
|  | Asian | 2 | 12.5 |
|  | Other and Mixed race | 4 | 25.0 |
| Religion | No religion | 13 | 81.25 |
|  | Christianity | 1 | 6.25 |
|  | Judaism | 2 | 12.5 |
| Education | Graduate/Postgraduate | 15 | 93.75 |
|  | Undergraduate | 1 | 6.25 |
| Country of Residence | Netherlands | 8 | 50.0 |
|  | Mexico | 8 | 50.0 |

#

# S.II Thematic Analysis of Qualitative Interviews

Participants reported a range of challenges during psychedelic ceremonies, including the emergence of past traumas, prolonged periods of anxiety or panic, intense grief, physical distress, and feelings of dissatisfaction with the experiences. Common subthemes among these challenges included a sense of impending danger, feelings of being trapped or alone, a loss of control, and significant physical discomfort.

Despite the inherently distressing nature of these experiences, participants noted various positive subsequent outcomes. Improvements in personal relationships (P3, P8, P10, P14), increased honesty and a greater willingness to disclose their inner selves (P10), and a heightened capacity for forgiveness (P3, P10, P12) were among the reported benefits. The challenging experiences themselves were often perceived as valuable "lessons" that prompted a renewed focus on self-care and psychological healing (P2, P4, P6, P8, P12, P13, P16). Participants recognised the importance of addressing unresolved grief or trauma (P2, P3, P10, P12), the value of seeking support (P16), and the need for emotional acceptance (P4). As one participant stated, "I believe the transformation I sought would not have been possible without the challenges I faced during the journeys" (P3).

Through our thematic analysis of participant accounts, particularly focussing on their responses to challenging experiences, we identified various specific responses and coping strategies deployed by participants. These responses were classified into four themes based on their relationship to participants' internal processing, embodied interaction with the environment, interpersonal engagement, and interventions initiated by facilitators. These themes align with current psychedelic research, which suggests that these substances facilitate connections with the self, the world, and others ([8]; [26]). Table 1 presents illustrative quotations from participants for each theme.

**Theme 1: Inner Responses**

Participants employed various introspective strategies, termed "inner responses," to navigate and address challenging experiences during psychedelic ceremonies. These strategies encompassed deliberate attentional focus, self-directed dialogue, and the creation of personal meaning.

1.1 Accepting and observing: Participants described attempts to relinquish control and adopt an observational stance towards their experiences, employing phrases such as "lean in," "let go," "accept," "surrender," and "observe" (P2, P3, P6, P8, P14, P15, P16). One participant stated, "...practising meditation beforehand helped me learn to observe thoughts without resistance. This allowed me to navigate the journey smoothly and gain different perspectives on the issue" (P14).

1.2 Self-talk, re-assurance, and "reminders to self": Participants engaged in internal dialogue to provide reassurance and motivation during challenging moments (P3, P4 P9). They reminded themselves of the therapeutic potential of their experience: "Feeling trapped and helpless can be terrifying... But then I would remind myself to trust in the healing power of the psychedelic and fully embrace the experience. It was there for a reason" (P3).

1.3 Interrogating and commanding the challenge: Some participants took a proactive approach by directly questioning their experiences and seeking to modify their subjective content (P5, P8). For instance: "When I encountered the giant spider, I asked it two questions, 'What are you trying to tell me?' 'Are you a medicine'? ... So, I took matters into my own hands and said, 'If you're not a medicine, you must leave now'" (P5).

1.4 Interpretation and meaning-making: Participants assigned symbolic or psychological significance to their distress (P2, P3, P8), akin to the "integration" phase in psychedelic therapy ([27]). As one participant described, "At that moment, I felt my heart break. I thought I was having a heart attack, but then I recalled our discussion on sacred surgery and realised this was symbolic" (P2).

1.5 Journaling: Journaling served as a means of externalising, giving structure to and verbalising the experience (P10, P12), signifying a transition to more behavioural and interpersonal responses. As one participant explained: "The subsequent three hours were devoted to fervent journaling while experiencing fits of laughter and tears... I reflected on my family and friends, contemplating the impact of this experience on my relationships" (P10).

**Theme 2: Embodied Practice and Engagement with the Environment**

Embodied practices and engagement with the environment emerged as crucial strategies for managing challenging experiences during psychedelic ceremonies. Participants employed a variety of techniques, highlighting the complexity and fluidity of their interaction with their surroundings and physical states.

2.1 Intentional use of breath: Breath served as a grounding element and a tool for maintaining a sense of safety during turbulent experiences (P3, P9, P12, P15, P16). For example: "I recalled what one of the guides mentioned during the preparation: to focus on my breath. I directed my attention to my breathing, and it helped me stay grounded and feel a sense of safety" (P15).

2.2 Opening the eyes and moving around (versus closing the eyes and remaining still): Participants reported varying degrees of physical movement during the experience, deviating from what is sometimes perceived as the “recommended” stance of stillness and eye closure. Physical movement and active engagement with the environment served as a bridge to navigate through challenges (P3, P6). One participant noted, "After taking the second dose, I decided to move around a bit. I let go of my expectations for a profound psychedelic experience and embraced the beauty of the ceremony" (P6).

2.3 Sensory engagement with the environment and participation in the ceremony: Immersing themselves in the sensory-rich context of the ceremony and the surrounding environment emerged as a crucial factor in managing distressing experiences (P3, P6, P10, P11). Participants found solace and distraction by appreciating the beauty of the ceremony, engaging in activities like dancing, and embracing the sights, sounds, and natural surroundings. One participant described the impact of sensory engagement, stating, "When I got up and started moving around, I was able to fully appreciate the beauty of the ceremony. It helped me shift my focus away from my disappointment... Dancing on the periphery of the ceremony space had the most profound impact" (P6).

2.4 Leaving the ceremony: In some instances, participants sought relief by temporarily stepping away from the ceremonial space (P2, P11). This distancing allowed them to find respite from overwhelming experiences: "I had been advised to embrace the difficult moments, with the understanding that they would pass. But they didn't. The intensity became unbearable. Towards the end, while others were dancing, I simply needed to step outside" (P2).

2.5 Sleep: As a coping strategy, some participants found solace in sleep, effectively disengaging from the immediate psychedelic experience, and allowing time for a transition to a more manageable state (P2, P4, P13). One participant shared, "I finally fell asleep around 3 AM, and when I woke up the next day, I felt like my normal self again" (P4)

**Theme 3: Interpersonal Responses**

This theme emphasises the role of social interactions in modulating the nature of challenging psychedelic experiences. Participants described a range of interpersonal responses, including avoidance of social interactions, seeking help, and engaging in candid disclosure or "confession."

3.1 Avoiding social interactions: For some participants, isolation and withdrawal from social interactions provided a coping strategy during challenging experiences (P4, P12). These individuals found it difficult to emotionally engage with others and preferred to be alone. One participant explained, "I tried to avoid interacting with others in the group because I couldn't emotionally connect with what they were saying, and I didn't want to hurt anyone's feelings. So, I retreated to my room" (P4).

3.2 Seeking help: In contrast, at other times participants actively sought support from fellow participants or facilitators to navigate their distressing moments (P12, P16). They reached out for assistance, often seeking comforting presence or guidance in regulating their experiences. This was not always easy: "[...] I found myself and my body in this state of fear with no way out. I stayed there for far too long and eventually mustered the courage to remove my eyemask and ask a facilitator for help" (P16).

3.3 Disclosure and "confession": Participants also described the relief and liberation they experienced by openly sharing their inner experiences with others (P3, P10). This act of disclosure served as a catharsis, allowing them to process their emotions and find support in the presence of understanding individuals. One participant narrated, "When I finally shared my story, I felt a weight lift from my body like never before. Tears of sadness turned into tears of joy, but there were still many tears!" (P10).

**Theme 4: Facilitator Responses**

This theme encapsulates the critical role of facilitators in assisting participants during challenging moments in psychedelic experiences. Facilitators provided expertise and timely interventions, encompassing a range of supportive actions from tactile reassurance to auditory grounding techniques.

4.1 Accepting Physical touch and reminders to breathe from facilitators: Facilitators utilised physical touch and provided guidance on the use of breath to help individuals navigate distressing episodes (P3, P12, P14, P16). These actions offered comfort and facilitated a sense of grounding: "The situation changed when a facilitator approached me, held my hand, stroked my head, and guided me to regulate my breathing. Through their support, I regained a sense of regular breathing and navigated my way out of a dark, dark place" (P16). As such thi subtheme entails the participant’s capacity and willingness to accept this kind of support.

4.2 Feeling Listened to : Facilitators played a crucial role in actively listening and holding space for participants during challenging moments (P2, P3, P10). For some participants this provided a non-judgmental and supportive presence, allowing individuals to express their experiences and emotions. One participant expressed gratitude, saying, "It took me 30 minutes to utter a single word, but the facilitator held my hand and waited patiently until I was ready. They 'held space' for me, as they say" (P10).

4.3 Reassurance: Facilitators offered reassurance to participants to alleviate anxiety (P2, P3, P11), which helped some participants feel safe and secure during distressing episodes. One participant recalled, "Internally, my mind was racing, and I remember sweating profusely. The lead guide reassured me, reminding me that I had consumed a significant amount of truffles and that I was safe and doing remarkably well. That reassurance was incredibly comforting" (P11).

4.4 Rhythm and sound: Facilitators used the power of music and rhythm as grounding techniques to support participants in navigating their experiences (P3, P10). They created an auditory environment that helped individuals find stability and move through challenging moments. A participant reflected, "The music accompanying our trip played a significant role in ensuring its beneficial nature, even during the most difficult times" (P10).

4.5 Introducing new elements: Facilitators introduced new elements into participants' experiences, such as objects (P2) or other individuals (P3), to facilitate shifts in perception and engagement. These introductions had a profound impact on participants' experiences and served as catalysts for transformation. One participant described the effect of a facilitator offering a rose quartz crystal, stating, "After hours of feeling very little... one of the facilitators came and handed me a rose quartz crystal. At that moment, I felt my heart break" (P2).

##

# S.III Challenges reported by participants

Of the 16 participants in the sample, 7 participants rated the experience as among the top 10 most challenging experiences in their life (and in two cases these participants had 2 such challenging experiences in separate ceremonies), and 2 participants reported that this experience was in the top 5 most challenging experiences of their life. None rated their experience as the single most challenging experience in their life.

In the interests of summarising the wide range of experiences, these are categorised below according to the dominant emotion or focus of distress for participants.

Note that in some cases participants reported on multiple different challenges from different psilocybin ceremonies during their stay at the psychedelic retreat.

|  | |
| --- | --- |
| Re-living a trauma memory or difficult episode from one’s past | Four participants reported re-living traumatic memories and associated physical sensations or emotional challenges. Interestingly, the two participants in the sample who rated their experience as amongst the top 5 most challenging in their lives were in this category. These included re-living memories of physical assault (P12), a medical emergency (P15), childbirth (P3), and emotional and sexual abuse in childhood (P10). |
| Anxiety and panic reactions | Anxiety or panic reactions were characteristic of many of the challenging experiences reported by participants in this sample.    In some cases anxiety was triggered by frightening images (e.g. of a large spider) (P5), sensations (e.g. of being buried alive) (P3), or catastrophising beliefs (e.g. belief that one is dying, or has gone “crazy”) (P11, P8). In one case, panic was a secondary reaction to feeling overwhelmed in the face of guilt and shame (P16). One participant (P14) also had an anxiety reaction leading to a brief fainting spell, from which he was nursed back by facilitators.    Some participants also ruminated on relational difficulties during their trips (P7, P10, P13), and one participant reported paranoid thoughts about the facilitators “experimenting” on him (P6). |
| Grief and sorrow | Prolonged feelings of grief and sorrow during the ceremony, in relation to feelings of rejection or loss of attachment figures (P2, P7), or with regards to the suffering of humanity at large (P8), were noted by a few participants in the sample. For one participant this was overwhelming and led to concerns of being “stuck” in the psychedelic state (P2). |
| Physical distress and discomfort | Participants reflected on the unpleasant somatic dimensions of their challenging experiences, some of which, but not all, might be understood as sympathetic nervous reactions in the context of anxiety (e.g. sweating, nausea), or in association with trauma memories (as noted above). These included feeling hot and cold (P6, P14), muscle cramps and tension (P6, P9), heaviness (P8), aches and pains (P3, P8, P12), nausea (P3, P8, P12), sweating (P8, P11), and hypersensitivity to sensations on the skin (P8, P14). In one case this discomfort continued after the psychedelic ceremony had ended (P12). |
| Disappointment and frustration in the context of an ‘underwhelming’ experience | In contrast to the physically or emotionally turbulent accounts noted above, a few participants also reported being challenged with frustration and disappointment in the face of “underwhelming” experiences (although in some cases went on to have more intense experiences in subsequent ceremonies). This involved difficulties “breaking through”, “concentrating” or having the “deep experience” they had hoped for (P7, P10, P11). |

#

# S.IV Outcomes reported by participants

Participants noted a variety of positive outcomes from their participation in the psychedelic ceremonies that they attributed at least in part, if not fully, to the challenging episodes they experienced. Several participants noted a sense of deep ‘connectedness’ consequent to their challenging experience (P8, P14), and other participants noted increased transparency and self-disclosure with others (P10), or increased ‘acceptance’, tolerance (P3) and forgiveness for others, including partners (P10, P12) and children (P3).

“As of today, I've never been happier in my life. My marriage is the best its ever been. Without these difficult episodes, the journey would have just been a fun time on mind altering substances. Instead, it was a life changing moment for me.” (P10)

“My relationship with both of my kids has been so different the past 14-15 months. I have only raised my voice twice, whereas it used to be every week or so. I play with them more.” (P3)

Many participants noted that their challenging experiences constituted a ‘lesson’ or allowed for important ‘realisations’, and led to renewed motivations to take care of oneself and engage in psychological healing. These included difficult realisations about needing to release ‘unprocessed’ grief or trauma (P2, P12), learning about the value of emotions, both good and bad (and not pushing them away) (P4, P8), and lessons such as the importance of managing expectations (P6).

“I realised that I needed to process the loss, the grief. I knew I was good at "clamping my wheels back on" - even running big global companies at Board level. But the grief was so deep and painful that I couldn't deal with it. It needed to come out.” (P2)

“I felt a bit cheated of the wonderful, blissful experience described by others who talk about their experiences. However, as someone who has struggled their entire life with intense emotions and struggled to control them, even to the point of wishing them away completely, I think this experience was incredibly important.” (P4)

Closely related were realisations about the need to let go of resentment, and what might be seen as a ‘victim identity’, as well as increased sense of responsibility and belief in the ability to control aspects of one’s emotional experience (or at least ‘reaction’ to it):

“Huge role it definitely showed me I needed to put me first and finally deal with my internal pain and no longer keep it pushed way down. Same with forgiving him ” (P12)

“They were the best teaching moments that really showed me where my work lay. Not in the experience but my reaction to it” (P13)

“I experienced that I have the will and the power to change a situation, a bad emotional situation. I understood that it is me who creates those emotions and bad experiences. And that it is also me, me who can change it.” (P8)

One participant who experienced panic as he faced his ‘shadow self’ noted that the main outcome of his challenging episode was learning to ask for help, as well as a sense of responsibility for needing to help himself:

“although this was the toughest part, it was probably the most healing. I learned a lot from this experience – I learned to ask for help and that at the end of the day only I can help myself internally” (P16)

In contrast, a few participants were less emphatic about the significance of their challenging experiences. One participant who experienced panic as he thought he was witnessing his own death noted that this challenge was only “moderately important” (P11). Another participant who was able to “dissolve” a disturbing image noted that he felt his challenge had been cut short, and had tried “too hard to protect myself” (P5).

#

# S.V Response to challenging psychedelic experience inventory (ReCiPE)

**Instructions:**

People respond in different ways when faced with challenging psychedelic experiences, and these responses can be more or less helpful.

How did you respond, during the psychedelic experience, when you were experiencing distress, difficulty or discomfort? And how helpful were these responses in managing, resolving or ending the challenging experiences you faced?

Below is a list of ways in which people may respond to challenging psychedelic experiences. Please indicate how helpful you found each response, or select 'N/A' if you did not engage in this behaviour.

**Scale**:

1. N/A (I did not try this)

2. Yes but it was not helpful

3. Yes and it was somewhat helpful

4. Yes and it was substantially helpful

**Items:**

When I experienced distress, difficulty or discomfort during the psychedelic experience:

1. I tried to let go, accept or surrender to the experience

2. I tried to observe my mind and not fight it

3. I addressed the challenge with a question (for e.g. ‘what are you trying to tell me?’)

4. I assertively told the challenging experience to “stop!” or “go away!”

5. I directed anger or aggression towards confronting my challenging experience

6. I was able to interpret the challenging physical sensations, images or visions as having a deeper emotional, psychological or spiritual source

7. I wrote in a journal or diary (or other personal document)

8. I focused on my breathing

9. I tried closing my eyes, lying or sitting down

10. I tried moving around or moving my body to shift the experience

11. I tried opening my eyes and looking around

12. I danced

13. I sang, chanted or hummed

14. I changed the music

15. I changed setting or location (e.g. moving from inside to outside)

16. I tried to engage with the natural environment (e.g. looking at the sky, listening to the sounds of the forest etc.) if available

17. I tried to fall asleep

18. I tried to avoid social contact so I could be alone

19. I asked for help from a guide, friend or other person present

20. I was able to disclose something intimate or personal about myself to somebody else

21. I shared honestly with somebody present about a difficult or traumatic episode from my life

22. I asked for forgiveness or apologised for something I had done

23. I was given physical touch (e.g. hand holding, stroking, hugging etc.)

24. Someone offered me reassurance that I would be ok, or was doing well

25. I called upon non-human entities, spirits or a deity for help during periods of challenge

26. I took another drug, or consumed alcohol

#

# S.VI Study 2 Participant Characteristics (N=529)

| **Category (N=529)** | **M** | **SD** | **Range** |
| --- | --- | --- | --- |
| Age | 37.71 | 10.88 | 18-73 |
|  | **Subcategory** | **N** | **%** |
| Gender | Male | 243 | 45.94 |
|  | Female | 256 | 48.39 |
|  | Other | 30 | 5.67 |
| Index Psychedelic Experience | 1 month ago or less | 44 | 8.32 |
|  | 1-12 months ago | 136 | 25.71 |
|  | 1-2 years ago | 100 | 18.9 |
|  | More than 2 years ago | 249 | 47.07 |
| Ethnicity | Black | 10 | 1.89 |
|  | Asian | 8 | 1.51 |
|  | South Asian | 16 | 3.02 |
|  | South East Asian | 3 | 0.57 |
|  | White | 391 | 73.91 |
|  | Hispanic | 56 | 10.59 |
|  | Arab | 15 | 2.84 |
|  | Other | 30 | 5.67 |
| Religion | No religion | 198 | 37.43 |
|  | Christianity | 27 | 5.1 |
|  | Islam | 9 | 1.7 |
|  | Judaism | 11 | 2.08 |
|  | Hinduism | 6 | 1.13 |
|  | Buddhism | 18 | 3.4 |
|  | Spiritual | 237 | 44.8 |
|  | Other | 23 | 4.35 |
| Psychedelic Consumed | 5-MeO-DMT | 13 | 2.46 |
|  | Ayahuasca | 48 | 9.07 |
|  | DMT | 17 | 3.21 |
|  | LSD / Acid | 144 | 27.22 |
|  | Mescaline / Peyote / San Pedro | 7 | 1.32 |
|  | Other | 5 | 0.95 |
|  | Psilocybin / Magic Mushrooms | 186 | 35.16 |
|  | Combination of Drugs | 109 | 20.6 |

# S.VII Frequency of responses for each ReCiPE item

|  | **Item** | **Did Not Attempt** | **Not Helpful** | **Somewhat Helpful** | **Substantially Helpful** |
| --- | --- | --- | --- | --- | --- |
| 1 | I tried to let go, accept or surrender to the experience | 35  (6.6%) | 46  (8.7%) | 169  (31.9%) | 279  (52.6%) |
| 2 | I tried to observe my mind and not fight it | 90  (17.%) | 30  (5.7%) | 179  (33.8%) | 230  (43.5%) |
| 3 | I addressed the challenge with a question (e.g., ‘what are you trying to tell me?’) | 235  (44.4) | 22  (4.2%) | 119  (22.5%) | 153  (28.9%) |
| 4 | I assertively told the challenging experience to “stop!” or “go away!” | 390  (73.7%) | 78  (14.7%) | 40  (7.6%) | 21  (4.0%) |
| 5 | I directed anger or aggression towards confronting my challenging experience | 444  (83.9%) | 35  (6.6%) | 36  (6.8%) | 14  (2.6%) |
| 6 | I was able to interpret the challenging physical sensations, images or visions as having a deeper emotional, psychological or spiritual source | 129  (24.4%) | 32  (6.0%) | 157  (29.7%) | 211  (39.9%) |
| 7 | I wrote in a journal or diary (or other personal document) | 343  (64.8%) | 14  (2.6%) | 73  (13.8%) | 99  (18.7%) |
| 8 | I focused on my breathing | 167  (31.6%) | 25  (4.7%) | 161  (30.4%) | 176  (33.3%) |
| 9 | I tried closing my eyes, lying or sitting down | 67  (12.7%) | 71  (13.4%) | 175  (33.1%) | 216  (40.8%) |
| 10 | I tried moving around or moving my body to shift the experience | 136  (25.7%) | 73  (13.8%) | 167  (31.6%) | 153  (28.9%) |
| 11 | I tried opening my eyes and looking around | 112  (21.2%) | 121  (22.9%) | 184  (34.8%) | 112  (21.2%) |
| 12 | I danced | 349  (66.0%) | 11  (2.1%) | 79  (14.9%) | 90  (17.0%) |
| 13 | I sang, chanted or hummed | 340  (64.3%) | 13  (2.5%) | 84  (15.9%) | 92  (17.4%) |
| 14 | I changed the music | 303  (57.3%) | 23  (4.3%) | 93  (17.6%) | 110  (20.8%) |
| 15 | I changed setting or location (e.g., moving from inside to outside) * | 237  (44.8%) | 30  (5.7%) | 131  (24.8%) | 129  (24.4%) |
| 16 | I tried to engage with the natural environment (e.g., looking at the sky, listening to the sounds of the forest etc.) if available | 198  (37.4%) | 27  (5.1%) | 102  (19.3%) | 202  (38.2%) |
| 17 | I tried to fall asleep | 371  (70.1%) | 107  (20.2%) | 38  (7.2%) | 13  (2.5%) |
| 18 | I tried to avoid social contact so I could be alone | 305  (57.7%) | 45  (8.5%) | 100  (18.9%) | 79  (14.9%) |
| 19 | I asked for help from a guide, friend or other person present ** | 268  (50.7%) | 28  (5.3%) | 99  (18.7%) | 133  (25.1%) |
| 20 | I was able to disclose something intimate or personal about myself to somebody else | 305  (57.7%) | 9  (1.7%) | 95  (18.0%) | 120  (22.7%) |
| 21 | I shared honestly with somebody present about a difficult or traumatic episode from my life | 353  (66.7%) | 8  (1.5%) | 66  (12.5%) | 102  (19.3%) |
| 22 | I asked for forgiveness or apologised for something I had done | 390  (73.7%) | 11  (2.1%) | 66  (12.5%) | 62  (11.7%) |
| 23 | I was given physical touch (e.g., hand holding, stroking, hugging etc.) | 260  (49.1) | 22  (4.2%) | 99  (18.7%) | 148  (28.0%) |
| 24 | Someone offered me reassurance that I would be ok, or was doing well | 214  (40.5%) | 25  (4.7%) | 134  (25.3%) | 156  (29.5%) |
| 25 | I called upon non-human entities, spirits or a deity for help during periods of challenge | 375  (70.9%) | 12  (2.3%) | 54  (10.2%) | 88  (16.6%) |
| 26 | I took another drug, or consumed alcohol | 432  (81.7%) | 33  (6.2%) | 48  (9.1%) | 16  (3.0%) |

*n=527

**n=528
